# Indeterminate and Enriched Propositions in Context Linger: Evidence From an Eye-Tracking False Memory Paradigm

**DOI:** 10.3389/fpsyg.2021.741685

**Published:** 2021-10-21

**Authors:** Caitlyn Antal, Roberto G. de Almeida

**Affiliations:** Department of Psychology, Concordia University, Montreal, QC, Canada

**Keywords:** compositionality, indeterminate sentence comprehension, propositions, eye-tracking, false memory, discourse context, pragmatics, semantic coercion

## Abstract

A sentence such as *We finished the paper* is indeterminate with regards to what we finished doing with the paper. Indeterminate sentences constitute a test case for two major issues regarding language comprehension: (1) how we compose sentence meaning; and (2) what is retained in memory about what we read in context over time. In an eye-tracking experiment, participants read short stories that were unexpectedly followed by one of three recognition probes: (a) an indeterminate sentence (*Lisa began the book*), that is identical to the one in the story; (b) an enriched but false probe (*Lisa began reading the book*); and (c) a contextually unrelated probe (*Lisa began writing the book*). The probes were presented either at the offset of the original indeterminate sentence in context or following additional neutral discourse. We measured accuracy, probe recognition time, and reading times of the probe sentences. Results showed that, at the immediate time point, participants correctly accepted the identical probes with high accuracy and short recognition times, but that this effect reversed to chance-level accuracy and significantly longer recognition times at the delayed time point. We also found that participants falsely accept the enriched probe at both time points 50% of the time. There were no reading-time differences between identical and enriched probes, suggesting that enrichment might not be an early, mandatory process for indeterminate sentences. Overall, results suggest that while context produces an enriched proposition, an *un*enriched proposition true to the indeterminate sentence also lingers in memory.

## Introduction

Sentences such as *Lisa began the book* are semantically indeterminate because they are not explicit about the event that the speaker intends to convey. As first discussed by Culicover (1970), in isolation, these sentences appear to carry an “infinite ambiguity” (p. 368). Although it is not clear what sort of activity Lisa began doing with the book, we seem to assign a default interpretation to this type of sentence—such as *Lisa began reading the book*. Indeterminate sentences have received considerable attention in the theoretical literature in cognitive science, often under the rubric “complement coercion” (e.g., Briscoe et al., 1990; Pustejovsky, 1995, 2011; Fodor and Lepore, 2002; de Almeida and Dwivedi, 2008; de Swart, 2012; Asher, 2015; de Almeida and Lepore, 2018). This phenomenon has been subject to numerous behavioral and neuroimaging studies (e.g., McElree et al., 2001; de Almeida, 2004; Pickering et al., 2005; Traxler et al., 2005; Pylkkänen and McElree, 2007; Husband et al., 2011; Katsika et al., 2012; de Almeida et al., 2016; Riven and de Almeida, 2021). The key issue under dispute is the nature of the linguistic and cognitive resources involved in resolving (or attempting to resolve) indeterminacy, pointing to two views of semantic composition. One is based on the lexical constituents and how they are combined syntactically, known as “classical” compositionality (e.g., Partee, 1995; Fodor and Lepore, 2002). The other, known as “enriched” compositionality (e.g., Pustejovsky, 1995; Jackendoff, 2002; Recanati, 2004), is based on the features of these lexical constituents and other so-called unarticulated or default constituents.

The dispute between enriched and classical views of compositionality touches on fundamental issues in cognitive science, including what kind of information concepts—the units of meaning representation—contribute to the propositions they partake. Numerous proponents of the enriched compositionality view hypothesize that a sentence such as *Lisa began the book* undergoes a process of “coercion,” which implies forcing the complement noun *book* to be interpreted as a default event performed with the book, thus yielding a proposition such as *Lisa began reading the book* (Pustejovsky, 1995, 2011; Jackendoff, 2002; Lapata et al., 2003; Traxler et al., 2005; Pustejovsky and Batiukova, 2019).^1^ Lexical meanings are thus said to be composed of properties (or *qualia* information) that contribute content to the resulting proposition—properties such as “serves for reading.” Classical compositionality holds the view that an initial proposition is faithful to the denotations of words and how they combine, with enrichment to this proposition only coming *via* pragmatic inferences computed from contextual or background information (Fodor and Lepore, 2002; de Almeida and Lepore, 2018). This is so because according to this later view, compositionality only holds if concepts do not decompose into features—mostly because there is no clear account of what features are, nor which features are to constitute the content of a concept—the problem of analyticity (Fodor, 1998; see also Quine, 1953, and de Almeida and Antal, 2021, for a recent discussion).

While there has been empirical support for both views (see de Almeida et al., 2016, for a review), only two studies have investigated the potential role of context in the process of resolving indeterminacy, with mixed results: one supporting classical compositionality (de Almeida, 2004) and another, the enriched view (Traxler et al., 2005). One reason for this discrepancy might be that in both studies contexts were relatively weak, containing two to three sentences, thus yielding results that might not directly speak to the potential effect of context over the proposition built from the indeterminate sentence. Moreover, most response-time and neuroimaging studies to date have shown that indeterminate sentences in isolation or in short contexts appear to take longer to process (e.g., McElree et al., 2001; Pickering et al., 2005; Traxler et al., 2005; Katsika et al., 2012), or engender different brain networks (Pylkkänen and McElree, 2007; Husband et al., 2011; de Almeida et al., 2016) compared to fully determinate sentences such as *Lisa read the book*. But it is not clear what is the very *source* of these effects—whether they are due to a mandatory semantic process such as coercion or to the triggering of pragmatic inferences beyond initial, *classic* composition.

A more recent study (Riven and de Almeida, 2021) attempted to address these potential shortcomings by investigating the role of strong contexts in the interpretation of indeterminate sentences, using a recognition memory paradigm (Sachs, 1967). Participants were presented aurally with contexts of about 60 words describing particular events such as someone wishing to begin reading a novel. Either immediately after an indeterminate sentence (e.g., *Lisa began the book*) or after 18s of neutral discourse, participants saw a probe sentence that was either (1) identical to the original indeterminate sentence (2) a contextually supported foil (*Lisa began reading the book*) or (3) a contextually unsupported foil (*Lisa began writing the book*). Participants’ task was to determine whether or not the probe sentence was identical to the one they heard embedded in context. At the immediate probe position, accuracy was close to ceiling (i.e., correctly accepting the indeterminate sentence and rejecting the foils). But at the delayed probe position, the contextually unsupported sentence was correctly rejected 90% of the time, with the indeterminate sentence and the contextually supported sentence both being correctly accepted or rejected about 50% of the time. Interestingly, the time to respond to these probes changed drastically with participants taking significantly longer to accept the indeterminate sentence than the biased foil at the late probe point. Riven and de Almeida suggested that indeterminate sentences are enriched over time, as a function of context, with participants later accepting *Lisa began reading the book* when they heard *Lisa began the book*. But, crucially, this study suggests that the proposition consistent with the original indeterminate sentence lingers in memory, against the view that sentences are enriched by mandatory, default semantic processes.

Our goal in the present study was twofold. First, we wanted to investigate the same phenomenon of contextual enrichment of indeterminate sentences by combining the memory paradigm employed by Riven and de Almeida (2021) with a more real-time reading measure of the probes. Second, we wanted to further determine the nature of the proposition obtained during the comprehension of the indeterminate sentence and its potential enrichment over time. To this end, we employed an experimental paradigm similar to the seminal (Sachs 1967, 1974) studies, while eye-tracking the probe sentences at two time points, aiming to test for the potential false recognition of enriched sentences in context. In addition to tracing the nature of the proposition obtained, we also wanted to find out if indeterminate sentences engender any processing cost, after sufficient information about the event had been provided by the context.

It should be noted that the influence of context on sentence *interpretation* has been amply demonstrated, and that this influence is subject to many textual and sentential variables.^2^ What is not clear, however, is the nature of the proposition that indeterminate sentences express: Do we initially obtain a proposition that is faithful to the *verbatim* form of the target sentence? Is this proposition enriched by mandatory processes such as coercion? Or is it enriched as a function of inferences computed from discourse? Beyond the classical Sachs effect, thus, we were interested on the nature of the proposition obtained over time—faithful or not to the original indeterminate sentence.

We made two sets of predictions, in line with the study by Riven and de Almeida (2021), which can be summarized as follows. Regarding probe recognition accuracy, we predicted that if indeterminate sentences are enriched by discourse rather than by default interpolation of semantic information, participants should accept true indeterminate probes and correctly reject contextually supported (henceforth, “enriched”) foils, at the immediate probe point. Over time, the false, enriched-probe proposition should effectively replace the true proposition conveyed by the original indeterminate probe, reflecting a high rate of false rejections of the indeterminate probe and false acceptance of the enriched probe. Regarding eye movement measures, we predicted that reading times on the post-verbal position of an indeterminate sentence (i.e., the complement noun phrase *the book*) would be attenuated by discourse information, but differing from the false enriched sentence probe only at the later probe time point. This would be demonstrated by differences obtained during second-pass reading times (i.e., re-reading), which are more susceptible to semantic processes than are first-pass reading times (see Rayner, 1998, for discussion).

## Materials and Methods

### Participants

Thirty-six Concordia University students (21 females; *M_age_*=24; *SD*=5) participated in the study. They were all native speakers of English and reported having normal or corrected-to-normal vision. All participants provided written informed consent and were treated in accordance with guidelines outlined by Concordia’s Human Research Ethics Committee.

### Materials

Twenty-four experimental passages (plus 24 fillers and 2 practice) from Riven and de Almeida (2021) were employed. They had the structure as in (1): the first three sentences formed a biasing context (1a), setting the stage for some event but without actually mentioning the verb that most likely named the event (e.g., *reading*). The fourth sentence contained the embedded indeterminate clause (1b), with the remaining sentences elaborating the neutral context (1c) (see Supplementary Materials S1).

(1)
  a. *Context:* Lisa had been looking forward to the new Grisham novel ever since it came out. She had finally managed to set aside some time this weekend and made sure to make her home library nice and cozy. First thing Saturday morning, Lisa curled up on the sofa in her library with a blanket and a fresh cup of coffee.
  b. *Indeterminate sentence:* With everything in place, Lisa began the book in her library.
  c. *Neutral passage:* Suddenly, the doorbell rang. Lisa grunted, put down her coffee and sluggishly made her way to the door. It was her neighbor John and he was out of peanut butter again. Looking through the cupboard, Lisa realized she was no better off. She told John he was out of luck and suggested he try calling Mary, their mutual neighbor.

During each trial, participants were presented with one of three types of recognition probes, as in (2), either immediately after the original indeterminate sentence or after some neutral discourse

(2)
  a. *Identical/Indeterminate*: Lisa began the book in her library
  b. *Contextually supported, enriched:* Lisa began reading the book in her library.
  c. *Contextually unrelated:* Lisa began writing the book in her library.

The verb inserted in the enriched sentence consisted of the verb most frequently listed during a fill-in-the-blank norming task (e.g., *Lisa began ______ the book*; Riven and de Almeida, 2021).

### Apparatus

The stimuli were presented on a ViewSonic 19” CRT monitor (model G90fb, 1,020×768 pixel resolution, 100-Hz refresh rate). Experiment builder (Version 1.10.1630, SR Research, Ottawa, Ontario) was used to present the stimuli and record responses. Participants’ eye positions were recorded using a head-mounted eye tracker (EyeLink II, SR Research). Although the stimuli were read binocularly, only the camera on the right eye recorded eye movements, at a sampling resolution of 1,000Hz. Participants were seated at a viewing distance of 60cm from the screen, and 1° of visual angle corresponded to approximately 3-to-4 characters. Stimuli were presented in black characters on a white background.

### Procedure and Design

**Figure 1:**
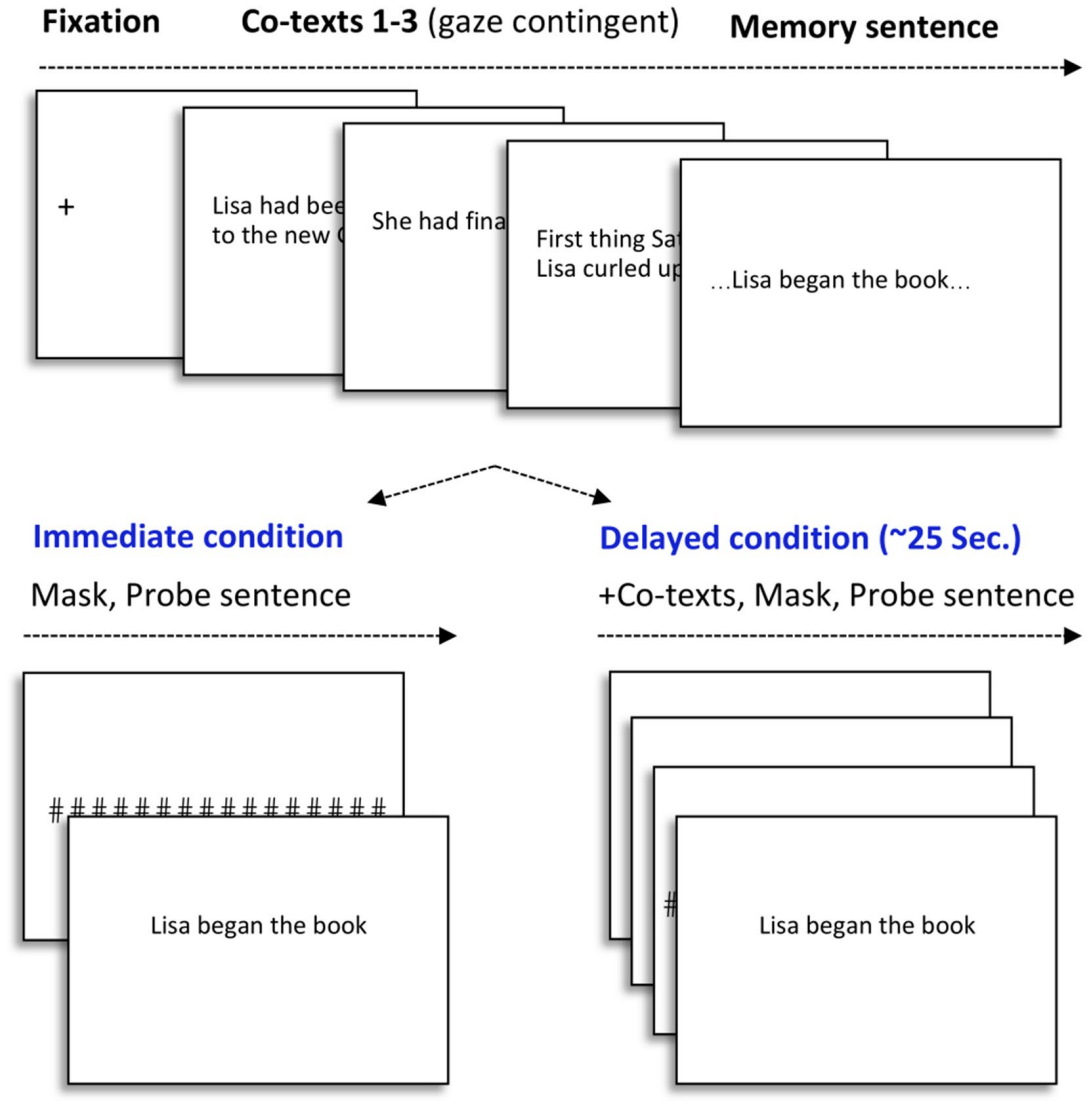
Schematic representation of each trial. Top row represents the presentation of the context, with the last screen representing the indeterminate sentence. The trial continues in one of two conditions, represented in the bottom row, either with an immediate presentation (0s) of one of the three probe sentences, or, in the delayed condition (+25s), with a neutral context, followed by one of the probe sentences.

Figure 1 represents the sequence of events in a given trial. Participants were instructed to read short stories, with sentences presented one at a time, and to press a button to initiate the next sentence. All trials began with drift correction, and all sentences within each trial appeared after a gaze contingent fixation cross for 120ms, located on the left-hand side of the screen, to ensure that participants read from left-to-right, from the beginning of each sentence. During each trial, one test sentence was presented for recognition. To notify participants that the test sentence would appear next, an acoustic signal was paired with a 200ms intervening mask (“#######”) that covered the length of the previous sentence. The test sentences occurred in one of two probe time points: immediate or delayed. In order not to disrupt participants’ natural reading, recognition probes were always presented once participants indicated having finished reading a sentence. In the immediate condition, the recognition probe was presented immediately after the indeterminate sentence. In the delayed condition, the probe was presented following an additional period of neutral discourse, corresponding to about 25s after the indeterminate sentence appeared in the story. Participants were instructed to respond, as quickly and as accurately as possible, whether or not the recognition probe presented on the screen had appeared word-for-word, in the passages of the story. If so, they were instructed to press the “yes” key, or to press “no” otherwise. The 24 experimental stories were counterbalanced among 6 lists, corresponding to 6 conditions (3 probe types, 2 probe points).

### Data Analyses

All participants’ overall accuracy to the recognition probes was above chance (i.e., above 50%). Further, participants’ responses that were two standard deviations above or below their respective means (4.22% of responses) were replaced by their upper or lower standard deviation cut-off tail values.

Recognition accuracy, measured in proportion of correct responses, was used as the dependent variable in a binomial generalized mixed-effects model. We also conducted a secondary analysis on recognition times (RTs), measured in milliseconds (ms), to investigate the decision difficulty associated with the three types of probe sentences at the delayed probe time. Given that the three sentences have different lengths, reading demands might differ across probe types. As such, we computed a variable to isolate the RTs associated with decision difficulty alone. Specifically, we subtracted from each observation in the delayed condition, the mean RT of the corresponding sentence in the immediate condition, the latter of which included only correct responses (e.g., (RT_enriched/delayed_) – mean (RT_enriched/immediate/correct_)). These RTs were used to objectively assess the degree of difficulty associated with long-term recognition for each sentence type (see Riven and de Almeida, 2021). We also conducted a third set of analyses based on eye-tracking measures. We analyzed first- and second-pass reading times (in ms) in the region of the noun phrase complement for all recognition probe sentences. We refer to first-pass reading time as the total duration of all fixations entering the complement noun phrase region (e.g., *the book*), provided that there were no fixations to words further down the sentence, that is, to the right of the complement region. This is often called gaze duration and is taken to reflect not only lexical access but also the integration of the word with its sentential context (Rayner, 1998). We refer to second-pass reading time as the total duration of all fixations when returning to the complement region, that is, re-reading time. This relatively late measure is taken to reflect greater effort in processing the information in the region, including reanalyses and other more strategic processes. Values of zero (i.e., instances where the region of interest was not fixated) were included in first- and second-pass analyses (Clifton et al., 2007). Based on predetermined cut-off values, we removed fixations shorter than 80ms and longer than 800ms (0.64% of fixations; Rayner, 1998).

We conducted linear mixed effects models (Baayen et al., 2008) using the *lme4* package (Bates et al., 2013) for the R statistical programming environment (Development Core Team, 2012; Core Team, 2014). For all analyses, probe time (immediate, delayed) and probe type (identical, enriched, unrelated) were entered as fixed factors. The models analyzed the effects of probe time and probe type on participants’ RTs and accuracy to recognition probes, as well as first- and second-pass reading times for the noun phrase complement region (e.g., *the book*) for all recognition probe sentences. All models included random intercepts for subjects and items, as justified by the likelihood tests. Our fully fitted models included random intercepts for participants and items, and the interaction between probe time and probe type as fixed effects. The baseline condition for all models consisted of the unrelated probe type at the immediate probe time, except for the RT change model which only included the unrelated condition as the baseline. We derived *p* values for all main effects and interactions using the Likelihood Ratio Test to compare the full model to a reduced model excluding the relevant term (Winter, 2013, 2019). Planned comparisons were conducted using the *emmeans* package with Tukey’s correction (Lenth et al., 2018), and using Type III sums of squares for analysis of variance model comparisons. Inspection of residual plots showed deviations from homoscedasticity and normality for second-pass reading times. As such, those analyses relied on square-root-transformed data.

## Results

### Recognition Accuracy

**Figure 2:**
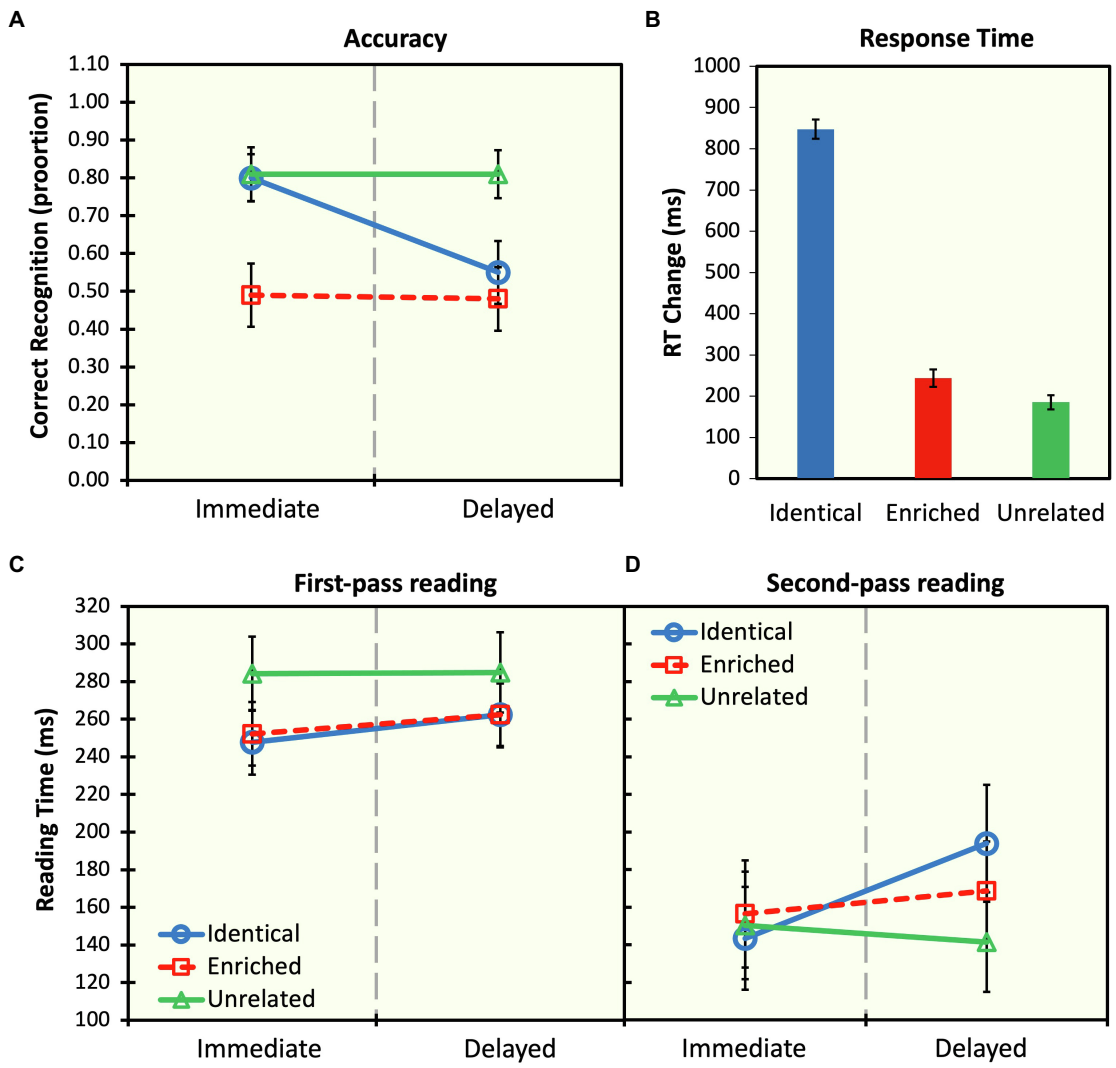
**(A)** Mean recognition accuracy for visual probe sentences shown at the offset of the original indeterminate sentence (immediate) and after about 25s (delayed) of neutral discourse for identical (e.g., *Lisa began the book*), enriched (e.g., *Lisa began reading the book*), and unrelated (e.g., *Lisa began writing the book*) sentences. **(B)** Mean change in response times (ms) from the immediate to delayed probe points for the three probe conditions showing how much more time was necessary to respond to the probe in the delayed condition, compared to the immediate condition. **(C)** First-pass reading times (ms) and **(D)** second-pass reading times for the complement noun phrase (e.g., *the book*) in the three probe conditions at the two probe points. Error bars represent one standard error of the mean.

The full model was compared to a null model consisting of only random predictors and was found to provide a statistically significant better fit to the data, *χ*^2^(5)=95.19, *p*<0.001. There were also significant main effects of probe time and probe type, and a significant interaction (see Table 1). At the immediate probe time position, participants recognized the identical probe with significantly greater accuracy than the enriched probe (*z-ratio=5.58, p*<0.001). However, at the delayed probe time position, this effect disappears (*z-ratio*=1.29, *p*=0.79), with both the identical and enriched probes being correctly recognized 55 and 48% of the time, respectively. Furthermore, results also showed that the identical probe is recognized with greater accuracy at the immediate time point, in comparison with the delayed time point (*z-ratio*=4.53, *p*<0.001). However, this difference was not found for the enriched probes (*z-ratio*=0.12, *p*=1.00; see Figure 2A).

**Table 1:** Top: logistic regression of recognition accuracy to the three probe types at the immediate and delayed probe time points. Bottom: Linear regression of response time change from immediate to delayed probe presentation.

*Accuracy*						
Predictor	β	SE β	z-value	p-value	95% CI of β	Null Comparison^*^
Intercept	1.52	0.23	6.48	< 0.001	[1.06, 1.98]	
Probe Time (Delayed)	0.03	0.31	0.09	0.93	[−0.58, 0.64]	*χ*^2^(3)=21.88, *p* <0.001
Probe Type (Identical)	−0.04	0.30	−0.13	0.90	[−0.64, 0.56]	
						*χ*^2^(4)=88.18, *p* <0.001
Probe Type (Enriched)	−1.58	0.28	−5.67	< 0.001	[−2.13, −1.04]	
Probe Time: Probe Type (Identical)	−1.28	0.42	−3.09	0.002	[−2.10, −0.47]	
						*χ*^2^(2)=14.17, *p<* 0.001
Probe Time: Probe Type (Enriched)	−0.06	0.39	−0.15	0.88	[−0.83, 0.71]	
**RT Change**						
**Predictor**	**β**	**SE β**	**t-value**	**p-value**	**95% CI of β**	**Null Comparison**
Intercept	139.55	182.97	0.76	0.45	[−219.08, 498.17]	
Probe Type (Identical)	681.54	177.60	3.84	< 0.001	[333.44, 1029.64]	*χ*^2^(2)=15.29, *p* <0.001
Probe Type (Enriched)	43.47	188.56	0.23	0.82	[−326.10, 413.04]	

* *Null comparisons refer to model comparisons for main effects (Probe Time and Probe Type) and interactions (Probe Time x Probe Type). Baseline for accuracy was the immediate unrelated condition. Baseline for RT change was the unrelated condition*.

Overall, recognition accuracy for the identical and enriched probes diminished with delay. In particular, the odds of correct recognition at the immediate time point were 1.14 times that of the delayed time point. Also, the odds of correct recognition for the unrelated probes were 1.19 times that of the identical probes. More importantly, the odds of correct recognition for identical sentences were 1.41 times greater than that of enriched probes.

### Recognition Time

To further investigate the processes underlying false memories, we analyzed RTs associated with delayed recognition. Specifically, we measured the increase in RT from baseline by subtracting mean RTs for correct responses in the immediate condition from the RTs of the corresponding delayed condition (see Riven and de Almeida, 2021). We then fitted a linear mixed-effects model to the RT data with probe type entered as a fixed effect and participants and items entered as random effects. The full model was compared to a null model consisting of only random predictors and was found to provide a statistically significant better fit to the data, *χ*^2^(2)=15.29, *p*<0.001. Mean RT change is presented in Figure 2B, and a summary of the linear mixed-effects analyses for RT change is presented in Table 1. Planned comparisons revealed that, with delay, participants were significantly slower in responding to identical probes than enriched probes (*t-ratio*=3.08, *p*=0.007) and unrelated (*t-ratio*=−3.82, *p*<0.001). In other words, responses to the identical probes engendered the greatest change in RTs across the two time points, in comparison with the enriched and unrelated probes.

### Eye-Tracking: First- and Second-Pass Reading Times

For first- and second-pass reading times, the full models were compared to null models consisting of only random predictors and were found to provide a statistically significant better fit to the data for first-pass reading, *χ*^2^(5)=16.91, *p*=0.005, and for second-pass reading, *χ*^2^(5)=12.06, *p*=0.04. As can be seen in Table 2, during first-pass reading, there was a main effect of probe type but no main effect of probe time and no interaction (see Figure 2C). Also, during first-pass reading, there was no difference in reading times between the identical and enriched probes, at both time points (immediate: *t-ratio*=−0.23, *p*=1.00; delayed: *t-ratio*=0.06; *p*=1.00). No differences between probes were also observed during second-pass reading times. Specifically, at both time points, there was no difference in reading time at the position of the noun phrase complement between the identical and enriched probes (immediate: *t-ratio*=−0.50, *p*=1.00; delayed: *t-ratio*=0.86, *p*=0.96). There were also no differences between the identical probes, across both presentation times (*t-ratio*=−2.59; *p*=0.10), but identical probes engendered marginally longer reading times than the unrelated probes at the delayed time point (*t-ratio*=2.72; *p*=0.07, see Figure 2D).

**Table 2:** Linear regressions for first-and second-pass reading times at the position of the noun phrase complement.

*First-Pass Reading*						
Predictor	β	SE β	t-value	p value	95% CI of β	Null Comparison^*^
Intercept	283.51	12.79	22.71	< 0.001	[258.44, 308.57]	
Probe Time (Delayed)	−0.03	11.87	−0.003	1.00	[−23.29, 23.23]	*χ*^2^(3)=2.17, *p* =0.54
Probe Type (Identical)	−35.35	11.73	−3.02	0.03	[−58.34, −12.37]	
						*χ*^2^(4)=15.49, *p* =0.004
Probe Type (Enriched)	−32.70	11.92	−2.74	< 0.006	[−56.06, −9.33]	
Probe Time: Probe Type (Identical)	13.94	16.79	0.83	0.41	[−18.97, 46.85]	
						*χ*^2^(2)=0.76, *p*= 0.69
Probe Time: Probe Type (Enriched)	10.61	16.82	0.63	0.53	[−22.35, 43.57]	
**Second-Pass Reading**						
**Predictor**	**β**	**SE β**	**t-value**	**p value**	**95% CI of β**	Null Comparison^*^
Intercept	8.91	0.82	10.81	<. 0.001	[7.30, 10.53]	
Probe Time (Delayed)	−0.31	0.97	−0.32	0.75	[−2.20, 1.59]	*χ*^2^(3)=8.37, *p* =0.04
Probe Type (Identical)	−0.20	0.98	−0.21	0.83	[−2.12, 1.71]	
						*χ*^2^(4)=8.00, *p* =0.09
Probe Type (Enriched)	0.28	0.99	0.29	0.77	[−1.65, 2.22]	
Probe Time: Probe Type (Identical)	2.86	1.38	2.07	0.04	[0.16, 5.57]	
						*χ*^2^(2)=4.32, *p* =0.12
Probe Time: Probe Type (Enriched)	1.53	1.38	1.11	0.26	[−1.18, 4.24]	

* *Null comparisons refer to model comparisons for main effects (Probe Time and Probe Type) and interactions (Probe Time x Probe Type). Baseline was the immediate unrelated condition*.

## Discussion

The goal of our study was to investigate the nature of the proposition obtained from an indeterminate sentence in context over time. We were particularly interested in determining whether the proposition that one encodes from an indeterminate sentence is enriched by default—i.e., by a semantic coercion operation—or whether it is affected primarily by context through pragmatic inferences computed from the local discourse. Methodologically, we employed offline and online measures by combining a discourse-based sentence probe recognition paradigm with eye-tracking to extend a classic false memory for “gist” effect (Sachs, 1967, 1974).

Regarding accuracy, we found that participants correctly accepted the identical probes at the immediate time point with high accuracy, but that this effect declined to chance level at the delayed time point—a similar effect as the one obtained by Sachs (1974)—with materials that substantially change the meaning (i.e., truth value) of the original sentence. These results are also similar to those obtained by Riven and de Almeida (2021), with the same materials but employing a different modality. We also found that participants falsely accepted the enriched probe at the immediate time point about 50% of the time, which is at odds with the previous study. This result may suggest that participants have enriched the indeterminate sentence during the earliest moments of its encoding. False acceptance of the enriched probes remained at chance for the delayed probe point, suggesting that the (enriched) proposition formed early during acquisition, within context, was stable. While the acceptance rates for the identical condition across both probe points are in line with the classical composition view, the incorrect acceptance rates for the enriched probe at the early probe point seem to support the enriched composition view: immediately after reading *Lisa began the book* subjects falsely accept *Lisa began reading the book*.

There are, however, three issues that prevent us from fully endorsing a coercion effect. First is the time course of events. Our technique does not allow for probing the earliest stages of semantic representation. Notice that our probes were shown only about 300ms after the complement noun *book*—which was the time the mask was on the screen. Moreover, the mask would only appear after participants pressed a button to indicate they had read the sentence in the context. This time could have been enough for *contextual* influence on probes.

A second issue pertains to the nature of the results obtained for the change in RTs to the three probe types at the delayed point. As Figure 2B shows, the identical probes engendered a significant increase in RTs in comparison with the enriched and unrelated probes. One interpretation for this pattern of results is that, given that participants enriched the indeterminate sentence by default, being presented with the identical probe sentence might cause some form of surprisal effect and thus requiring a reanalysis of the sentence before making a decision. However, the change in RTs may also suggest that the proposition expressed by the identical probe remains viable at the delayed time point, effectively creating competition between alternative interpretations—that is, between the original and enriched propositions. Presumably, in the case of enriched probes, participants inferred that the enriched event—say, *reading*—occurred in the discourse and judged that they had acquired this information in the sentence that was originally presented to them in discourse. But why should this decision take longer for identical probes compared to enriched probes, especially since the two probe types yielded equal levels of accuracy at the delayed time point? We propose that there are additional inferences associated with the identical probes due to contextual information. In other words, the denotational representation of the indeterminate sentence presented in discourse during acquisition interferes with the contextually favored enriched interpretation at the delayed point. Thus, the original indeterminate proposition is never overwritten or replaced by the enriched one; rather, both propositions linger in long-term memory over time. In fact, other studies have also found evidence for multiple propositions, some consistent with a *literal* (i.e., based on an explicit, lexical-semantic denotation of sentence constituents) and another enriched or *non-literal* interpretation (e.g., Pissani and de Almeida, 2021). Similarly, studies investigating the so-called garden-path sentences have found results consistent with the idea that propositions compatible with multiple interpretations are held in memory (see, e.g., Christianson et al., 2001).

A third, related point is that by having presented participants with the enriched probe at the immediate point, we may have induced uncertainty and created a false memory of the true proposition. That is, given that the enriched probe was contextually supported, this led to competition between the identical and enriched propositions. In other words, when presented with the enriched probe, participants had to decide between the true, original proposition and the false proposition created by the enriched probe. This could account for why participants falsely accept the enriched probes at the immediate time position with about 50% accuracy, but have no problem correctly accepting the identical probe. While it could be the case that falsely accepting the enriched probe at the immediate position suggests that semantic enrichment occurs as a default process, the overall nature of our offline results seems to be more in line with the idea that *both* the original and enriched propositions linger in long-term memory. This position is consistent with a view of false memory representations that takes false and true propositions to co-exist and compete during recognition (Reyna et al., 2016; Riven and de Almeida, 2021).

The results from online eye-tracking measures also suggest similar interpretations. Specifically, if enrichment was a default process, we would have expected longer reading times in the region of the noun complement in the identical probe during first-pass reading. However, we found no difference between the identical and enriched probes at both the immediate and delayed positions during first-pass and second-pass reading. We should highlight that our most robust measures are that of gaze duration (that is, first-pass reading) because they are said to reflect not only lexical access but also processes of lexical integration (Rayner, 1998)—presumably semantic composition. Our second-pass measures, which reflect higher processes of interpretation, are not as robust because as it often happens during reading, there is a high percentage of trials in which there is no rereading involved (in the present study, 40.1%). Our results are in part consistent with those of Traxler et al. (2005) who also did not find gaze duration effects in the noun complement region. This lack of statistical difference in first- and second-pass reading suggests that if enrichment occurs, it might not be an automatic and encapsulated process.

Our results, however, do not reflect the moment-by-moment enrichment as participants *first* encounter the indeterminate sentence in context, but rather how those sentences are encoded over time. In order to evaluate the effect of context on the potentially immediate enrichment of indeterminate sentences, it would be important to extend the present study by embedding all three sentence types in the context. This would be a natural extension of the present study, given the inconsistent results of context effects (de Almeida, 2004; Traxler et al., 2005) on the time-course of enrichment. The present design, however, allowed us to determine the memory trace for indeterminate sentences soon after their encoding (immediate point) and about 25s later (delayed point).^3^ This manipulation showed that false memories for enriched foils are created soon after their encoding—an effect which was also found by Riven and de Almeida (2021).

## Conclusion

The results from both the online and offline measures employed in the current study suggest that both the original and enriched propositions linger in long-term memory. Thus, enriched meaning may not be built by default but instead might occur beyond the initial sentence composition *via* contextually driven pragmatic processes.

## Data Availability Statement

The raw data supporting the conclusions of this article will be made available by the authors, without undue reservation.

## Ethics Statement

The studies involving human participants were reviewed and approved by Concordia University Human Research Ethics Committee. The participants provided their written informed consent to participate in this study.

## Author Contributions

CA and RdA conceived the study, designed the experiment, and wrote the manuscript. CA programmed the experiment and conducted the statistical analyses. The final manuscript is the result of a team effort, by both CA and RdA. Both authors contributed to the article and approved the submitted version.

## Funding

This study was supported by grants from the Natural Sciences and Engineering Research Council of Canada (NSERC) and from the Social Sciences and Humanities Research Council of Canada (SSHRC) to RGdA, and fellowships from NSERC and SSHRC to CA.

## Conflict of Interest

The authors declare that the research was conducted in the absence of any commercial or financial relationships that could be construed as a potential conflict of interest.

## Publisher’s Note

All claims expressed in this article are solely those of the authors and do not necessarily represent those of their affiliated organizations, or those of the publisher, the editors and the reviewers. Any product that may be evaluated in this article, or claim that may be made by its manufacturer, is not guaranteed or endorsed by the publisher.

## Footnotes

^1^In the sense used here, a “default” interpretation is one which results from the most prominent *quale* for a particular verb-noun complement being interpolated into the semantic structure of the sentence. Thus, as Pustejovsky (1995, p. 88) proposed in his analysis of constructions such as *Mary enjoyed the movie*, “Although there are certainly any number of ways of enjoying something, our understanding of these sentences is facilitated by default interpretations of properties and activities associated with objects. The qualia of an object can be seen as the initial points from which to construct interpretations that would otherwise be ill-formed.” See also Lapata et al., 2003, and Pustejovsky, 2011, for similar approaches.

^2^See, e.g., Garrod and Sanford (1994). Also, it is not under dispute here that readers generate different types of inferences during text reading (see O’Brien and Cook, 2015, for a recent review), nor that what subjects keep in memory over time is something like the “gist”—or the propositional representation—of a sentence in context, rather than its verbatim representation (Sachs, 1967, 1974; Kintsch, 1974). Additionally, it has been demonstrated that subjects “pragmatically normalize” (Fillenbaum, 1974) and generate “pragmatic implications” (Brewer, 1977; Brewer and Sampaio, 2006) over time from sentences they hear or read.

^3^It is important to note that our experiment uses recognition memory as a tool to investigate propositional content because there cannot be recognition without comprehension as, arguably, there cannot be comprehension without propositional encoding (barring other forms of encoding such as imagery). That is, responses to probes go *pari passu* with their comprehension: with responses reflecting the degree to which representations obtained during encoding and probe match each other.
